# Molecular stress and neurovascular injury in the diabetic retina

**DOI:** 10.1172/JCI200945

**Published:** 2026-03-02

**Authors:** Chuanyu Guo, Akrit Sodhi

**Affiliations:** Wilmer Eye Institute, Johns Hopkins University School of Medicine, Baltimore, Maryland, USA.

## Abstract

Diabetic retinopathy (DR), the most common microvascular complication in patients with diabetes mellitus (DM), is a leading cause of vision loss worldwide. Sustained hyperglycemia plays a central role in promoting DR. However, tight glycemic control does not prevent — and indeed sometimes worsens — DR, highlighting the importance of ongoing studies aimed at improving our understanding of this complex disease. Over the last few decades, the dogma that DR is a vascular disease that results in secondary neuronal injury has evolved, as emerging evidence suggests that neurodegeneration occurs in parallel with or prior to vascular cell injury in the retina of patients with DM. This has led to appreciation of DR as a neurovascular disease, characterized by microvascular injury and neurodegeneration, both of which contribute to vision loss. Here, we explore how molecular stress (i.e., glucose dysregulation, dysmetabolism, oxidative stress, and inflammation) promote retinal vascular cell and neuronal injury in patients with DM. We focus on how these processes influence, and are influenced by, genes regulated by the HIF family of transcription factors in glial, vascular, neuronal, and inflammatory cells, with the goal of identifying new therapeutic avenues for the prevention or early treatment of patients with this vision-threating disease.

## Introduction

Diabetic retinopathy (DR), the most frequent microvascular complication in patients with diabetes mellitus (DM) (1), is diagnosed based on retinal microvascular changes observed on clinical exam, and categorized into four progressive stages: mild, moderate, and severe nonproliferative diabetic retinopathy (NPDR), and proliferative diabetic retinopathy (PDR) (2). Diabetic macular edema (DME), the accumulation of interstitial fluid within the macula, is a consequence of vascular hyperpermeability and may occur in any of these stages, but is more common in patients with more advanced DR (3). Despite the introduction of therapies targeting VEGF, a key molecule that promotes the retinal microvascular changes observed in patients with DM, DR remains a leading cause of vision loss in the working-age population worldwide (4). Developing effective therapies that delay or prevent DR is therefore an important goal in optimizing the care provided to patients with DM.

Clinical trial results consistently support a role for early and intensive glucose regulation to reduce the onset and progression of diabetic eye disease (5). Accordingly, early studies into the etiology of the vascular changes observed in patients with DR focused on the role of glucose/metabolic dysregulation in vascular cell health and function (6). This work led to an appreciation for the role of oxidative stress, prior to the development of overt ischemia, in the development of DR (7). Parallel studies implicated inflammation in microvascular injury in DR (8). Along with anatomical changes from DME and PDR, inflammation and injury to the retinal microvasculature, in turn, were thought to result in neuronal dysfunction and vision loss (8).

Since the physical manifestations of DR are all directly related to blood vessel pathology, the leading hypothesis was that DR is caused by the damaging effects of high blood sugar on the retinal microvasculature. However, more recent evidence suggests that early neuronal dysfunction in the retina can occur in parallel with, or even precede, detectable microvascular changes, leading to the current understanding of DR as a complex neurovascular disorder where the primary injury affects the integrated functional unit of neurons, glial cells, and blood vessels (9). In this Review, we discuss our current understanding of this complex disease, focusing on how in retinal cells, expression of the HIF family of transcription factors is influenced by (or influences) metabolic dysfunction, oxidative stress, and chronic inflammation. Furthermore, we explore how HIFs regulate the expression of the genes that affect the development of DR.

## Clinical changes in DR

### NPDR.

**Figure 1:**
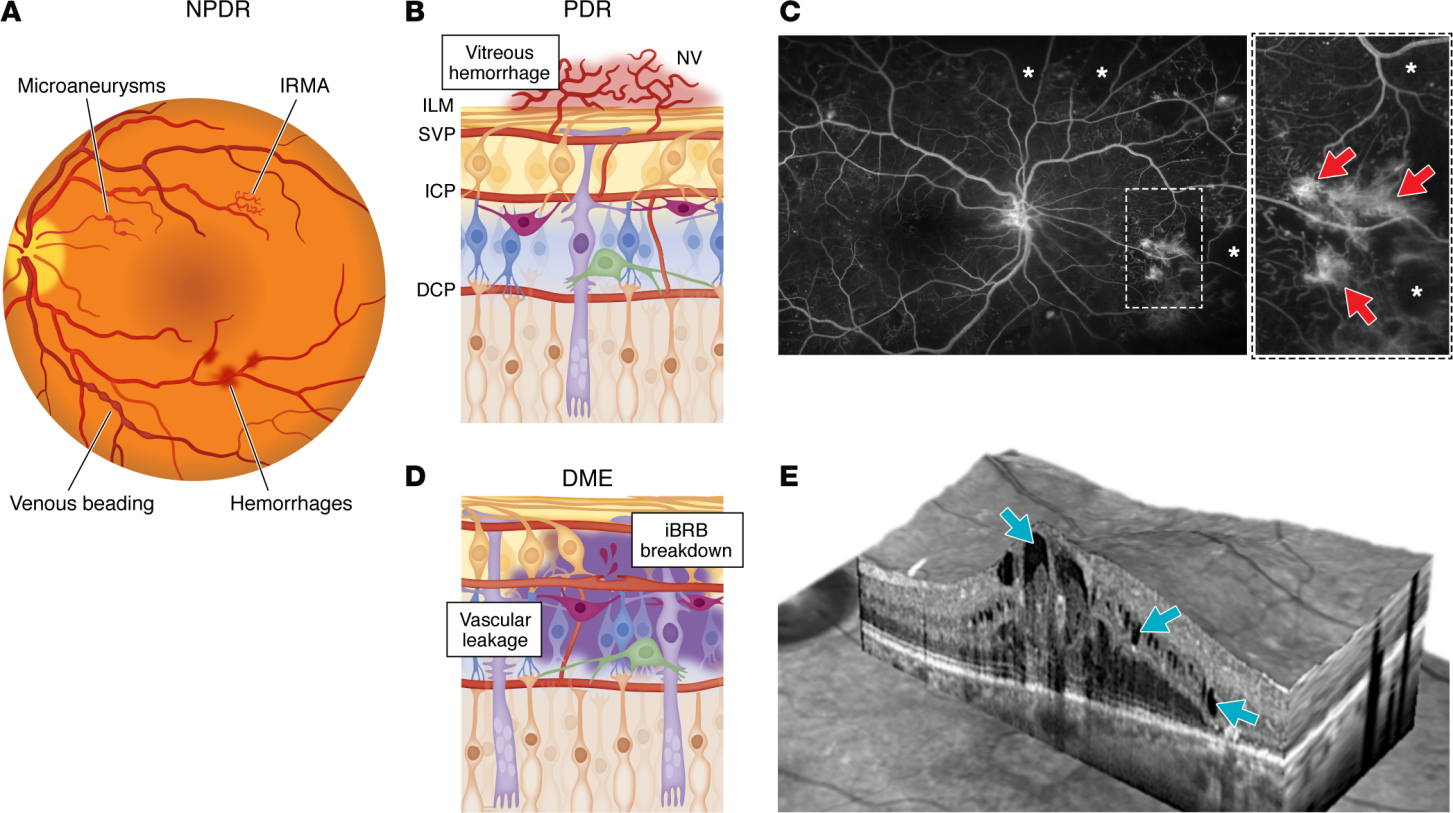
Retinal structure changes in diabetic retinopathy. (**A**) Schematic diagram of the microvascular changes observed in NPDR. Intraretinal hemorrhages, microaneurysms, venous beading, and/or IRMA occur during the progression from early to moderate and severe NPDR. (**B**) Schematic diagram of PDR. Development of retinal NV occurs when newly formed abnormal blood vessels from the superficial vascular plexus (SVP) penetrate the ILM and enter the vitreous and develop into fibrovascular tissue. ICP, intermediate capillary plexus; DCP, deep capillary plexus. (**C**) Fluorescein angiogram from a patient with PDR. Inset shows fluorescein leakage from retinal neovascularization (red arrows). Areas of capillary drop out (nonperfused retina) are indicated by white asterisks. (**D**) Schematic diagram of DME. Breakdown of the iBRB results in the leakage of intravascular fluid and circulating lipids and proteins into the extravascular space of the neurosensory retina. (**E**) Spectral-domain optical coherence tomography from a patient with DME demonstrating loss of the foveal contour and the accumulation of intraretinal fluid (blue arrows) in the inner and outer retina. Patient images were obtained with informed consent from an IRB-approved clinical study at the Johns Hopkins University School of Medicine.

In patients with mild NPDR, retinal microaneurysms and intraretinal hemorrhages are observed on clinical exam (Figure 1A). Progression to moderate and severe NPDR occurs with the development of extensive intraretinal hemorrhages, microaneurysms, venous beading, and/or intraretinal microvascular abnormalities (IRMA). At the cellular level (discussed below), these features involve glial cell activation, the dropout of pericytes and vascular smooth muscle cells (VSMCs), injury and death of capillary vascular endothelial cells (vECs), thickening of capillary basement membranes (BMs), leukostasis, vascular occlusions, and retinal ganglion cell (RGC) loss (10).

### PDR.

The development of retinal neovascularization (NV) (Figure 1B) and overt retinal ischemia (as seen on fluorescein angiography; Figure 1C) herald the development of PDR. In PDR, severe hypoxia disrupts the equilibrium between angiogenic and antiangiogenic factors, leading to pathological angiogenesis and the development of retinal NV. These newly formed abnormal blood vessels penetrate the internal limiting membrane (ILM), enter the vitreous, and develop into fibrovascular tissue. As it matures, the fibrovascular tissue can contract, leading to vitreous hemorrhage and/or tractional retinal detachment (11). If left untreated, PDR and its subsequent complications can lead to profound vision loss (11).

### DME.

Fluid balance in the retina relies on maintaining an equilibrium between the hydrostatic and oncotic pressure gradients across the retinal capillary beds (12). While the former functions to propel fluid out of the vessel into the interstitium, the latter arises from the concentration of proteins in the blood column, which holds fluid within the capillaries. In DME, breakdown of the inner blood-retinal barrier (iBRB) results in the leakage of vascular fluid (Figure 1D) and circulating lipids and proteins into the extravascular space of the neurosensory retina (13). If sufficient leakage occurs, this can result in macula edema (as seen on optical coherence tomography or OCT; Figure 1E), disrupting the normal retinal architecture and causing significant vision loss. DME may occur in both NPDR and PDR, but is more common in patients with more advanced DR (3). Nonetheless, DME and PDR are independent clinical features, with only about 30% of PDR patients exhibiting concurrent DME (14). Whereas PDR often responds robustly to even a single anti-VEGF injection, only 18%–45% of patients with DME achieve a meaningful improvement in vision despite ongoing treatment with anti-VEGF therapy (15), highlighting the heterogeneous and multifactorial nature of the disease.

## Cellular changes in DR

### Activation of glial cells.

**Figure 2:**
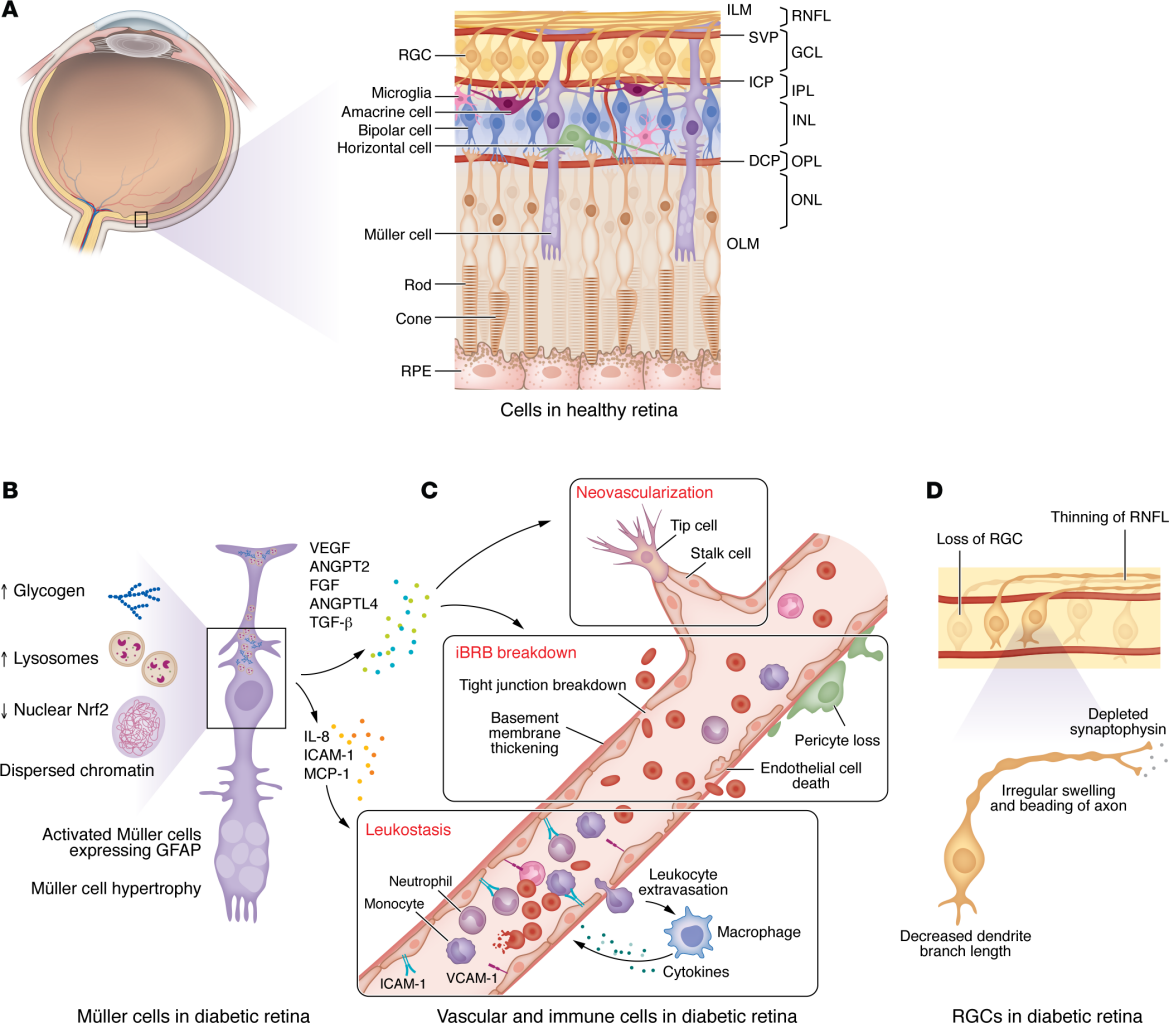
Retinal cellular structural changes in diabetic retinopathy. (**A**) Schematic diagram of the cellular structures in a healthy neurosensory retina. There are three layers of retinal blood vessels, the SVP, intermediate capillary plexus (ICP), and deep capillary plexus (DCP) located in GCL, IPL, and OPL, respectively. Müller cells span the entire retina and interact with both neurons and vascular cells. While the somas are located in the INL, the processes extend apically surrounding inner segments of photoreceptor cells and basally approaching the vitreal surface, forming OLM and ILM, respectively. (**B**) In the diabetic retina, activated Müller cells exhibit hypertrophy, increased expression of GFAP, nuclear deformation, chromatin dispersion, decreased nuclear Nrf2, and increased cytoplasmic glycogen and lysosomes. Hyperglycemia stimulates the secretion of vasoactive mediators from activated Müller cells, including VEGF, ANGPT2, FGF, ANGPTL4, and TGF-β, and inflammatory cytokines, including IL-8, ICAM-1 and MCP-1, thereby promoting vascular permeability and neovascularization, stimulating retinal fibrosis, and recruiting leukocytes, ultimately contributing to chronic inflammation and neurovascular degeneration in DR. (**C**) Vascular cell changes, including loss of retinal pericytes, vascular endothelial cell dysfunction and death, tight junction breakdown, basement membrane thickening, and leukostasis, lead to iBRB breakdown, vascular occlusion, and neovascularization. Monocytes and neutrophils are the principal leukocyte populations that drive leukostasis. Leukocyte extravasation and iBRB breakdown mutually reinforce one another in retinal inflammation and DR, while infiltrating monocyte-derived macrophages further amplify leukostasis through the release of cytokines. (**D**) DR affects the morphology, function, and survival of RGCs. These changes include thinning of the RNFL, reduced RGC dendritic field sizes, irregular swelling and beading of axons, deceased branching frequency, and depleted synaptophysin.

In the retina, there are three major types of glial cells: Müller cells, astrocytes, and microglia (16). These three cell types originate from distinct precursors, distribute to different areas of the retina, and exhibit varying morphologies, but they perform numerous overlapping functions within the retina under physiologic and pathological conditions. Müller cells constitute approximately 90% of the retinal glia, and are the most well-studied glial cell contributing to DR (16). Müller cell somas are located in the inner nuclear layer (INL) but their processes extend anteriorly to the vitreous surface where they form the ILM, and posteriorly to the outer retina where they surround the inner segments of photoreceptor cells to form the outer limiting membrane (OLM) (Figure 2A) (16). Müller cells provide structural support and nutritional factors, maintain water and ion homeostasis, regulate neuronal activity, and eliminate neuronal debris and glutamate, thereby influencing the function of both neurons and vascular cells throughout the neurosensory retina (17).

Glial fibrillary acidic protein (GFAP) is a characteristic molecular marker for both Müller cell injury and astrocytes (16). While GFAP is not expressed in healthy Müller cells, studies from postmortem eyes of diabetic patients and animals demonstrate increased GFAP expression prior to the development of overt vascular changes, suggesting that Müller cell activation may be an early event in the development of DR, one that precedes its clinical vascular hallmarks (18). Müller cells in diabetic rats demonstrate substantial subcellular morphological alterations, including deformed and denser nuclei, dispersed nuclear chromatin, and increased cytoplasmic glycogen, dense bodies, and lysosomes; these changes are particularly pronounced adjacent to capillaries as DR progresses (19) (Figure 2B). Similarly, immunohistochemical studies on human retinas from patients with early-stage DR demonstrate that the BM of retinal vessels adjacent to Müller cells are thickened, with embedded translucent round vacuoles and densely packed granules (20). Hyperglycemia stimulates Müller cells to secrete vasoactive mediators, including VEGF (21), FGF (22), angiopoietin-like 4 (ANGPTL4) (23), and TGF-β (24). These growth factors play crucial roles in promoting vascular permeability, regulating NV and contributing to the development of retinal fibrosis in DR (Figure 2B).

Unlike neuroectoderm-derived Müller cells and astrocytes, microglia originate from yolk sac erythromyeloid progenitors and migrate into the retina during late embryonic development (25). As resident macrophages, microglia are primarily localized within the inner and outer plexiform layers (IPL and OPL, respectively), where they exhibit distinct functional properties tailored to each microenvironment (25). Activated microglia have been implicated in all stages of DR. In NPDR, perivascular microglia cluster within the inner retinal layers; these cells exhibit moderately enlarged (hypertrophic) bodies and a loss of their typical organized alignment along the optic nerve (26). As the disease progresses to PDR, microglial cells migrate toward ischemic zones, where they specifically aggregate around newly developed, dilated vessels (27). Microglia execute functions that are distinct and nonredundant from those of infiltrating monocyte-derived macrophages in DR (28). While adaptive microglial responses may help eliminate toxic waste with relatively low pathogenic cytokine expression, maladaptive responses can result in the recruitment of infiltrated monocyte-derived macrophages that promote DR progression (28).

### Loss of retinal pericytes.

Pericytes are elongated stellate-shaped cells with finger-like processes that envelop capillary walls alongside vECs. Pericytes play a vital role in maintaining the vascular integrity of the iBRB (29). The retina has a pericyte-to-vEC ratio of approximately 1:1, higher than in any other tissue in the body (30). A bidirectional communication between pericytes/VSMCs and vECs plays a crucial role in preserving the integrity and function of retinal blood vessels (6). Pericytes/VSMCs control vEC proliferation and bolster the survival and integrity of the endothelium (31), while vECs release vasoactive agents (e.g., PDGF-B) to support the survival of pericytes/VSMCs (31). Pericytes further help protect retinal vECs from inflammation-triggered apoptosis by inhibiting the proliferation of activated T cells (32).

Progressive pericyte loss occurs in early DR and can be detected histologically by the formation of pericyte ghost vessels: vacant space containing remnants of pericytes sequestered within the capillary BM (Figure 2C) (33). Pericyte depletion leads to disruption of vasodilatation and promotion of vEC proliferation, primarily due to the reduced pericyte production of TGF-β (34). Pericyte loss also promotes endothelial inflammation and microglia activation, sensitizes ECs to VEGF-A with ANGPT2, and drives sustained angiogenic and inflammatory signaling (35–37). Although less recognized, the demise of arterial and arteriolar VSMCs is also observed in both diabetic animal models and patients with DM (38). These changes contribute to the emergence of microaneurysms and intraretinal hemorrhages (39), which are among the earliest clinically observable vascular changes in NPDR. Progressively, the capillary dilation, microaneurysms, and increased vascular permeability induced by pericyte and VSMC dysfunction contribute to vascular leakage, resulting in DME (40).

### vEC dysfunction and death.

Retinal vECs are the main components of the iBRB, a single-layer physical barrier separating the vascular lumen from the retina (41). Under hyperglycemic conditions, vECs are directly exposed to elevated glucose levels, leading to damage of vEC junctional properties, increased permeability, and vEC loss (41) (Figure 2C). Paradoxically, DM expedites the regeneration of vECs within the retinal microvasculature (42); this is speculated to cause vECs to exhaust their replicative lifespan and reach their Hayflick limit prematurely (43). The death of vECs eventually results in the formation of acellular capillaries: BM tubes without vEC nuclei that have a reduced diameter compared with normal capillaries (44). Acellular capillaries are observed in the retinas of long-term diabetic animals and in postmortem retinal tissue from patients with DM (38).

### Leukostasis and vascular occlusion.

Leukocytes are immune cells that distribute throughout the body, including the blood and lymphatic system. In early DR, leukocyte activation (a consequence of increased expression of inflammatory mediators) in the setting of reduced retinal blood flow causes leukostasis: adherence of leukocytes to the vascular endothelium (Figure 2C) (45). Monocytes and neutrophils are the major leukocytes driving leukostasis (46, 47). Leukostasis can occlude retinal capillaries, contributing to the development of capillary nonperfusion and ischemia (40), a precursor for retinal NV in PDR (see below). Due to leukocytes’ inherent capacity to generate toxic superoxide radicals, leukostasis also contributes to pericyte loss and vEC death, leading to leukocyte extravasation that allows activated leukocytes (monocytes and neutrophils) to infiltrate the retina, further worsening vascular permeability and capillary nonperfusion observed in DME and DR (48).

### RGC injury.

DR affects the morphology, function, and survival of many retinal neurons, but RGCs appear to be particularly vulnerable to injury in the diabetic retina. A significant thinning of the retinal nerve fiber layer (RNFL) in patients with DM has been detected through scanning laser polarimetry, consistent with RGC loss (49). This was later corroborated using spectral-domain OCT (SD OCT) (50). The RNFL, ganglion cell layer (GCL), and IPL are thinner in diabetic patients with no DR (51), minimal DR (52), and mild DR (53) compared with age-matched (nondiabetic) controls, suggesting that injury to RGCs precedes clinical evidence of vascular changes in the retina. The ganglion cell–inner plexiform layer (GC-IPL) thickness also decreases faster in diabetic patients (with or without DR) compared with nondiabetic controls, and the decrease in inner retinal thickness correlates with duration of DM and DR progression (54).

These studies have been corroborated in multiple diabetic rodent models, in which thinning of the NFL and GCL has been reported as early as 4 weeks after diabetes onset (44, 55). By 3 months, decreased numbers of RGCs and increased TUNEL and cleaved caspase-3 staining were observed in the GCL of diabetic animals, suggesting that loss of RGCs occurs through apoptosis (56). Consistent with these observations, histological staining of retinas from patients with DM have also demonstrated RGC loss and increased expression of proapoptotic markers, including BAX, FAS, and cleaved caspase-3 and -9 (57, 58).

Similar to other neurodegenerative diseases, the structural changes observed in patients with DM are accompanied by early and sustained retinal dysfunction, particularly RGC dysfunction, including in patients with minimal or no microvascular changes (59). In patients with DM without DR (or with very early DR), functional impairments have been reported in contrast sensitivity, perimetry testing, and dark adaptation, and can be detected by multifocal electroretinogram (mfERG) (60, 61). These observations have been corroborated in diabetic mice, in which decreased RGC function is observed as early as 15 days after induction of hyperglycemia (62).

Reduced RGC dendritic field sizes, irregular swelling and beading axons, and deceased branching frequency have been reported in postmortem studies of diabetic retinas (63) (Figure 2D). Accordingly, both maximum and average dendrite branch length of RGCs were significantly decreased in mice as early as 2 weeks after induction of hyperglycemia (62). A decrease in the quantity of axons within the optic nerve is also observed in diabetic animals (64). This may represent a modification in the dendritic architecture of neurons and help explain the reductions in IPL thickness in DM patients and animal models (44, 56).

### Photoreceptor damage.

In the outer retina, the survival and function of photoreceptors (rods and cones) are also affected by DM. Degeneration of the outer segments of rods, most M-cones, and some S-cones are detected in the retina of patients with DM (65). Images using adaptive optics also reveal a reduction in cone cell density among individuals with advanced stages of DR (66). Similarly, in 3-month-old diabetic *Ins2^Akita^* mice, a 10% loss of S-cones has been reported (67). Functional studies further corroborate rod and/or cone deficits in patients with DM, prior to overt signs of vascular cell injury (68).

## Pathological processes contributing to DR

The molecular mechanisms underlying DR, particularly vascular dysfunction and retinal neurodegeneration, can be broadly categorized into three groups: dysmetabolism, oxidative stress, and chronic inflammation.

### Dysmetabolism.

**Figure 3:**
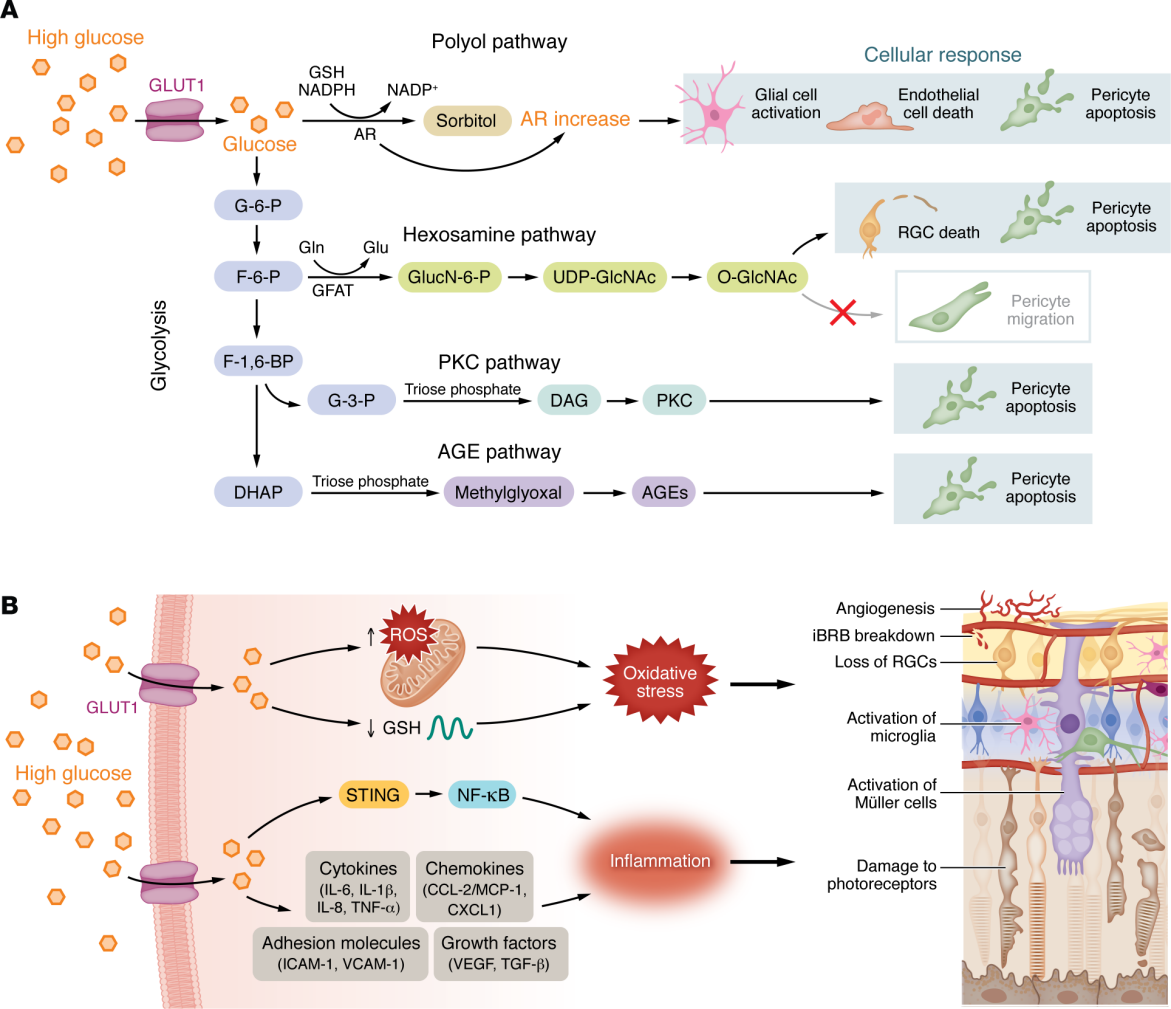
Pathological process in diabetic retinopathy. (**A**) Schematic diagram of dysmetabolism in response to high glucose. Aldose reductase (AR), a key enzyme in the polyol pathway, converts glucose into sorbitol, a highly hydrophilic sugar alcohol that is difficult to metabolize and accumulates in cells, contributing to glial cell activation, pericyte apoptosis, and endothelial cell death. Uridine diphosphate–N-acetylglucosamine (UDP-GlcNAc), produced via the hexosamine biosynthetic pathway, is the donor for O-GlcNAcylation, which mediates hyperglycemia-induced RGC death, impairs pericyte migration, and promotes retinal pericyte apoptosis. Increased protein kinase C (PKC) activity in retinal endothelial cells in diabetic retina induces pericyte apoptosis and acellular capillaries. AGEs stimulate pericyte apoptosis, angiogenesis, and breakdown of the iBRB. (**B**) High glucose enhances ROS accumulation while suppressing GSH expression in the retina, leading to oxidative stress and injury to the retinal microvasculature and neurons, including angiogenesis, iBRB breakdown, loss of RGCs, activation of Müller cells, activation of microglia, and damage to photoreceptors. High glucose also upregulates STING, which initiates the expression of inflammatory genes through NF-κB. In patients with DR, ocular tissues exhibit elevated levels of proinflammatory cytokines (IL-6, IL-1β, IL-8, TNF-α), chemokines (CCL-2/MCP-1, CXCL1), adhesion molecules (ICAM-1, VCAM-1), and growth factors (VEGF, TGF-β), which further promote injury to the retinal microvasculature and neurons.

Glucose homeostasis is elegantly regulated by coordinated interaction of glycolysis, the citric acid cycle, and oxidative phosphorylation. Glycolysis is a highly conserved metabolic pathway that converts glucose to lactate through a series of exquisitely synchronized enzymatic reactions. The concentration of glycolytic intermediates is tightly regulated. Prolonged exposure to high glucose results in increased production of glycolytic intermediate metabolites that accumulate and can contribute to the development of DR when shunted into four potentially damaging pathways: the polyol pathway, the hexosamine pathway, the protein kinase C (PKC) pathway, and the advanced glycation end products (AGEs) pathway (69) (Figure 3A). The accumulation of the metabolic intermediates from those four pathways affects glial cells, pericytes, endothelial cells, and RGCs, leading to angiogenesis, breakdown of the iBRB, and RGC death in DR (Table 1).

### Oxidative stress.

Reactive oxygen species (ROS) are continuously produced in all cells to facilitate cellular processes. Low levels of (or transient increases in) ROS can promote vEC regeneration and growth (70). The main physiologic source of ROS is the electron transport chain in mitochondria, but they are also generated from NAD(P)H oxidase (Nox), nitric oxide synthases, and cytochrome P450 (71). Oxidative stress occurs when there is an imbalance between the generation and elimination of ROS. The retina is uniquely vulnerable to oxidative stress as it is constantly exposed to visible light and UV radiation, leading to continuous generation of ROS. The outer retina is also highly metabolically active and therefore continuously under high oxygen tension from the underlying (high flow/high oxygenation) choriocapillaris. The polyunsaturated fatty acids that comprise photoreceptor outer segment membranes are also readily oxidized (72). In DM, this is further exacerbated as the high levels of circulating serum glucose further increase superoxide levels (73) and decrease activity and levels of one of the main cellular antioxidants, glutathione (GSH) (74). Müller cells serve as the primary reservoir of GSH (75). In response to tissue stress, Müller cells release GSH to provide support to other cells in defense against oxidative challenges (76). Nuclear factor erythroid 2-related factor 2 (Nrf2), a key transcription factor and regulator of cellular antioxidant defense, is also preferentially expressed in Müller cells (77). In cultured Müller cells, hyperglycemia causes a rapid reduction in nuclear Nrf2 (78), which may be mediated by the REDD1/GSK3 pathway (79). Accumulation of ROS in the diabetic retina ultimately results in chronic oxidative stress and contributes to impairment of biological macromolecules, metabolic abnormalities, and injury to the retinal vasculature and neurons, contributing to DR (80) (Figure 3B).

### Chronic inflammation.

The retina is an immune-privileged tissue protected by the BRB, an immunosuppressive microenvironment, and intrinsic defenses such as microglia and the complement system (81, 82). In early DR, when the BRB remains intact, microglia and the complement system are mildly activated in the retina and function to clear intraretinal metabolic intermediates and maintain homeostasis. In advanced DR, as immune privilege becomes compromised, infiltrating immune cells including monocyte-derived macrophages enter the retina, driving chronic inflammation and contributing to vascular and neuronal injury (82).

Hyperglycemia stimulates Müller cells to secrete the inflammatory cytokine IL-8, which recruits leukocytes and amplifies diabetic retinal inflammation (83). In addition, activation of CD40, which is highly expressed in the retina of diabetic mice (84), leads to an increase in the expression of ICAM-1 and MCP-1 by Müller cells, further promoting the recruitment of leukocytes to retinal blood vessels and contributing to neurovascular degeneration (85). Accordingly, therapies targeting inflammatory pathways are used in the clinic to treat patients with DR, including those who respond inadequately to therapies targeting VEGF (86).

Recent evidence implicates the cyclic GMP-AMP synthase–stimulator of interferon genes (cGAS/STING) pathway in detecting cytosolic DNA and initiating the expression of inflammatory genes through NF-κB (87). STING is upregulated in patients with DR and in animal models of diabetic ocular disease, and contributes to the pathogenesis of DR by promoting retinal endothelial cell senescence and capillary degeneration (88). In addition, a series of proinflammatory cytokines (IL-6, IL-1β, IL-8, TNF-α), chemokines (CCL-2/MCP-1, CXCL1), adhesion molecules (ICAM-1, VCAM-1), and growth factors (VEGF, TGF-β) are all increased in ocular tissue from patients with DR and are believed to participate in the vascular and neuronal injury that characterizes DR pathogenesis (8) (Figure 3B).

### Interplay among these pathways in DR.

Oxidative stress and dysmetabolism pathways mutually reinforce each other to promote DR progression. The components and products from dysmetabolism pathways (Table 1) promote production of ROS. In turn, ROS amplifies the dysmetabolism pathways. Similarly, hyperglycemia-induced oxidative stress exerts a multifaceted influence on the immune response within the retina. For example, through mutual regulation, NF-κB signaling and ROS stimulate the release of inflammatory mediators involved in DR development (89). Oxidized lipids and proteins can also recruit microglia, which subsequently initiate the innate immune response to remove oxidative products (82). A common consequence of oxidative stress in the retina is mitochondrial injury, which leads to the release of mitochondrial DNA into the cytosol (90). This, in turn, activates cGAS/STING signaling, which exacerbates oxidative damage and stimulates immune system activation (91). Oxidative stress also inhibits expression of complement factor H, thereby affecting regulation of the complement system in the retina (92) while simultaneously boosting formation of AGEs (93), which serve as persistent antigenic stimuli that induce proinflammatory cytokine production (94).

## HIFs and HIF-regulated genes in DR

There are several molecular pathways that are important for the development of DR. However, one family of transcription factors, the HIFs, plays a central role in glucose dysregulation, dysmetabolism, oxidative stress, and chronic inflammation, and contributes to many of the pathological outcomes observed in patients with DR described above.

### HIF-1α and HIF-2α.

**Figure 4:**
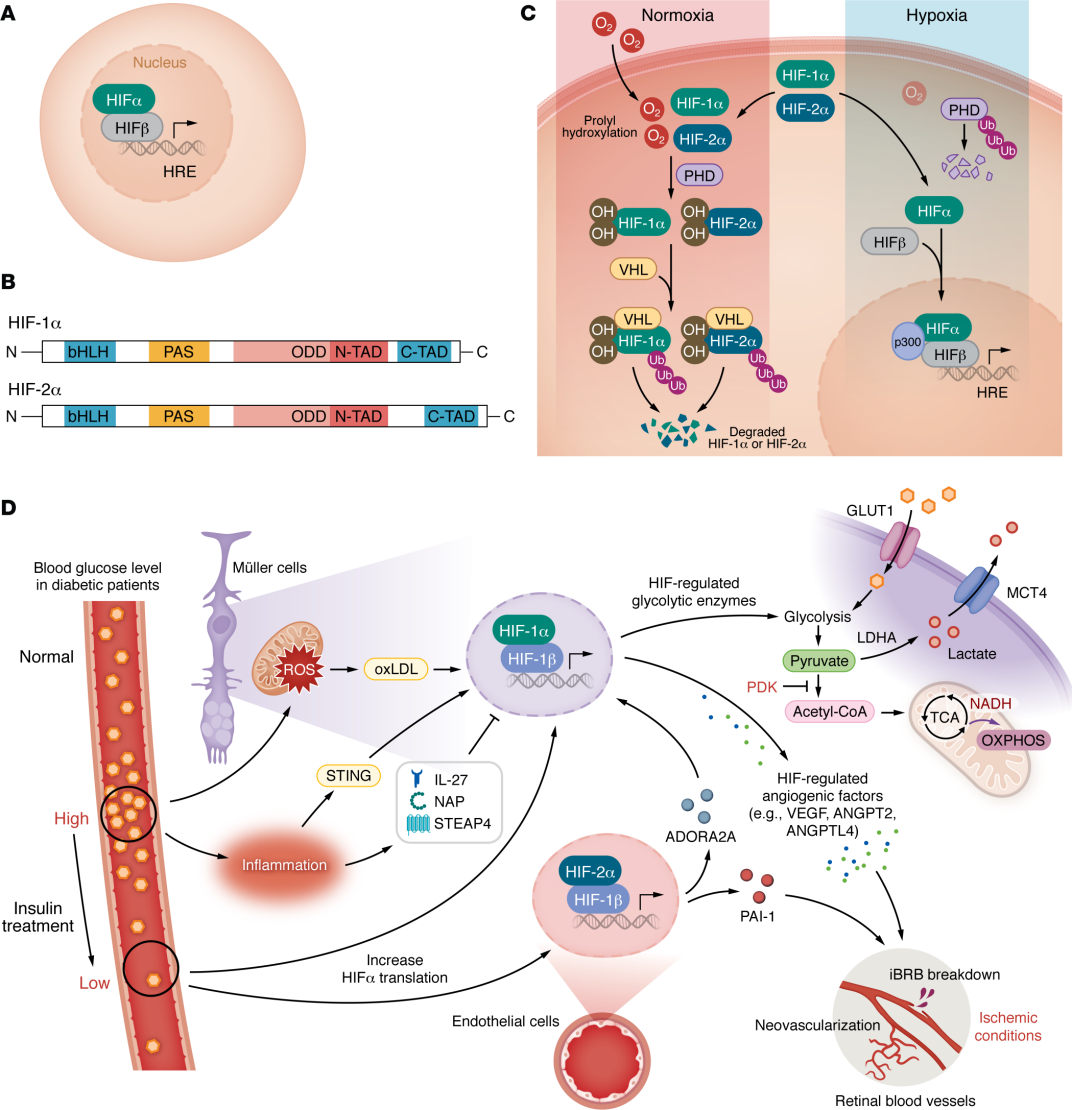
The interplay between HIFs and dysmetabolism, oxidative stress, and inflammation in diabetic retinopathy. (**A**) HIFs are heterodimeric proteins composed of an oxygen-sensitive α subunit and a ubiquitously expressed β subunit that bind to the hypoxia response element (HRE) of hypoxia-inducible genes. (**B**) HIF-1α and HIF-2α both contain a basic helix-loop-helix (bHLH) domain, PER-ARNT-SIM (PAS) domain, an oxygen-dependent degradation (ODD) domain, and an N-terminal and C-terminal transactivation domain (NTAD and CTAD, respectively). (**C**) Under normoxic conditions (left), HIF-1α and HIF-2α are hydroxylated at conserved proline residues by PHDs, marking them for recognition and degradation by the pVHL complex. Under hypoxic conditions (right), PHDs fail to hydroxylate HIF-1α and HIF-2α, allowing the proteins to accumulate, translocate to the nucleus, and activate transcription of their downstream target (hypoxia-inducible) genes. (**D**) In the diabetic retina, hyperglycemia stimulates oxidative stress and inflammation, stimulating accumulation of HIFs in retinal cells. Treatment with insulin can result in transient hypoglycemia that promotes increased translation and nuclear translocation of HIFs, independently of the canonical posttranslational modifications of HIFs observed in response to hypoxia. In Müller cells, accumulation of HIFs in response to hypoglycemia results in increased expression of GLUT1 and glycolytic enzymes, which promote glycolysis and lactate production. The lactate is exported through monocarboxylate transporter (MCT4) to support retinal neurons’ metabolism. However, in the diabetic retina, this physiologic response can have pathologic consequences, as increased HIF-regulated vasoactive mediators (e.g., VEGF, ANGPT2, and ANGPTL4) are also secreted from Müller cells in response to hypoglycemia. These mediators stimulate breakdown of the inner blood-retinal barrier, vessel leakage, and pathological angiogenesis. In endothelial cells, increased HIF-2α promotes expression of plasminogen activator inhibitor 1 (PAI-1) and ADORA2A. PAI-1 stimulates vascular leakage and angiogenesis, while ADORA2A induces HIF-1α accumulation, further supporting endothelial cell glycolysis and thereby promoting retinal neovascularization.

HIFs are heterodimeric proteins consisting of an oxygen-sensitive α subunit and a ubiquitously expressed β subunit (Figure 4A) (95). HIF-1α exhibits widespread expression, while HIF-2α is characterized by tissue-specific expression patterns (95). Human HIF-1α and HIF-2α share just 48% overall amino acid identity, but have very high similarity in their functional domains, including their DNA binding domain (83% identity) and oxygen-dependent degradation domain (70% identify). Accordingly, both HIF-1α and HIF-2α play important roles in regulating gene transcription in hypoxic cells (Figure 4B).

Under normoxic conditions, HIF-1α and HIF-2α are hydroxylated by prolyl hydroxylases (PHDs) at conserved proline residues (96), and subsequently recognized and degraded by the von Hippel-Lindau protein (pVHL) complex (Figure 4C) (97). In hypoxia, PHD activity is reduced due to lack of oxygen as a cosubstrate, leading to accumulation of HIF-1α and HIF-2α (98) (Figure 4C). HIFs directly influence the expression of over 1,000 genes, but only a subset of these are increased (or decreased) in response to hypoxia (99), and expression of these genes varies depending on the environmental stimulus, timing, and cell type.

### HIFs in DR.

Increased expression of both HIF-1α and HIF-2α has been reported in serum from patients with NPDR (100), vitreous (101) and ischemic retinal tissue (102, 103) from patients with PDR, as well as other ischemic retinopathies (104, 105) and in the ischemic retina of preclinical models (23, 103, 105–107). Accumulation of HIFs in the ischemic retina promotes the expression of the vasoactive mediators that drive retinal NV in PDR (108) and vascular hyperpermeability in DME (23). However, emerging evidence implicates accumulation of HIFs earlier in the development of DR, prior to the overt development of retinal ischemia. Accumulation of HIF-1α has been reported in the inner retina of patients with NPDR (23), while studies in diabetic mice have demonstrated that hyperglycemia for as little as one month, prior to evidence of retinal ischemia, is sufficient to result in accumulation of both HIF-1α and HIF-2α, as well as increased expression of vasoactive mediators they regulate (107).

It has recently been reported that transient episodes of hypoglycemia, a common occurrence in patients with DM undergoing tight glycemic control (TGC) or those with high glycemic variability, can also increase HIF-1α activity in inner retinal cells independently of its canonical regulation by posttranslational stabilization (Figure 4D) (109). Accumulation of HIF-1α was found to play a key physiologic role in maintaining the health of retinal Müller cells in hypoglycemia by influencing aerobic glycolysis and NAD^+^ and lactate production through its regulation of the expression of glucose transporter GLUT1 as well as key glycolytic enzymes, including lactate dehydrogenase-A (LDHA) and pyruvate dehydrogenase kinase 1 (PDK1) (109). However, this physiologic response to hypoglycemia has also been shown to have a paradoxical pathological consequence in the diabetic retina by upregulating expression of HIF-regulated vasoactive genes (109) that, in turn, promote breakdown of the iBRB and retinal vascular leakage in diabetic mice (110) (Figure 4D). These studies suggest that pharmacologic inhibition of HIFs could be a rational therapeutic avenue to prevent worsening of DR observed in patients initiating TGC (111).

Studies using tissue from patients with DR and diabetic mice implicate activated Müller cells as critical players in the HIF-1–dependent expression of vasoactive mediators (23). In Müller cell–specific *Hif1a*-knockout mice, there is a reduction in vascular leakage and prevention of NV despite persistence of retinal ischemia (112). HIF-2α expression in mouse models of ischemic retinal disease is observed in inner retinal cells as well as vECs (105) (Figure 4D). Pharmacologic or genetic inhibition of HIF-1α or HIF-2α accumulation in the inner ischemic retina prevents the development of retinal NV in a mouse model of ischemic retinopathy (105, 107). Collectively, these studies further support the use of therapies targeting both HIF-1α and HIF-2α to prevent or treat DR.

The critical role of HIFs in DR is further supported by the observation that therapies targeting one HIF-regulated vasoactive mediator, VEGF, are currently the gold standard for the treatment of both DME and PDR (108). However, in addition to VEGF, many other vasoactive genes regulated by HIFs also engage in the pathogenesis of DR, including VEGF receptor 2 (or KDR) (113), angiopoietin 2 (ANGPT2) and its downstream effector (114), vascular endothelial–protein tyrosine phosphatase (VE-PTP) (115), PDGF-B (116), ANGPTL4 (23, 117-119), MMPs (106), and plasminogen activator inhibitor-1 (PAI-1) (103). These proteins contribute to the development of vascular hyperpermeability in patients with DME (Table 2) and/or retinal NV in patients with PDR (Table 3). The recent introduction of therapies targeting ANGPT2 demonstrates a potential advantage of targeting HIF-regulated vasoactive mediators, in addition to VEGF, for the treatment of DR (120).

### HIFs and metabolism in DR.

The neurosensory retina is among the highest-energy-demand tissues in mammals (121). To produce ATP, retinal neurons mainly rely on glucose (122), predominantly metabolized via glycolysis despite the presence of oxygen (123). Similarly, retinal vECs produce over 80% of their ATP through aerobic glycolysis (124), and its inhibition reduces vEC proliferation and migration (125). After crossing retinal vECs, glucose can access other cell types through GLUT1 transporters located in cell bodies of Müller cells, and the outer segments of photoreceptors (126). Müller cell energy production is also dependent on glycolysis (127). Lactate produced by glycolysis in Müller cells is converted to glycogen and stored or released and transferred to retinal neurons, which convert lactate to pyruvate for oxidative phosphorylation (127). While 99% of glucose taken up by Müller cells is used for glycolysis (128), in conditions of metabolic stress (e.g., hypoglycemia), glycogen in Müller cells is broken down to produce essential metabolites (e.g., lactic acid) which are utilized by retinal neurons lacking sufficient resources (128).

Retinal photoreceptors exhibit greater metabolic activity compared with the inner retinal cells, but also undergo aerobic glycolysis to meet their high metabolic demand (129). Glucose used by photoreceptors is transported from the choroidal vasculature via the retinal pigment epithelium (RPE) (129). To support glycolysis in photoreceptors, RPE has limited glucose consumption. Instead, RPE generates ATP from oxidative phosphorylation utilizing nutrients from the bloodstream and metabolic by-products (e.g., lactate) derived from photoreceptors and other retinal neurons (129).

HIFs promote glycolysis in part by increasing expression of glucose transporters, such as GLUT1 and GLUT3 (130), thereby enhancing glucose uptake. They also augment expression of enzymes involved in glycolysis (109, 131), including hexokinases, phosphofructokinases, aldolases, glyceraldehyde 3-​phosphate dehydrogenase, phosphoglycerate kinase 1, enolases, and pyruvate kinase M. Furthermore, HIFs promote the expression of LDHA, to increase conversion of pyruvate to lactate, and monocarboxylate transporter 4, to export lactate to the extracellular space, and they increase PDK1 to block the conversion of pyruvate to acetyl-CoA, thereby inhibiting the TCA cycle (131) (Figure 4D).

Regulation of key glycolytic enzymes by HIFs has previously been shown to influence the development of pathological NV in mouse models of ischemic retinopathy (132). It was recently reported that HIF-2α mediates hypoxia-induced expression of the adenosine A2a receptor (ADORA2A) in human retinal vECs (133). This in turn induces HIF-1α accumulation and further promotes glycolysis (133). vEC-specific deletion of *Adora2a* decreases glycolysis and reduces NV in the retina from mice with oxygen-induced retinopathy (133) (Figure 4D).

### HIFs and oxidative stress in DR.

Although direct evidence demonstrating that HIFs regulate oxidative stress in patients with DR is still lacking, ROS production is known to activate HIFs in other disease contexts, including cancer (134). In human retinal vECs, the oxidative stress inhibitor scutellarin decreased high-glucose-induced ROS production that led to HIF-1α degradation, thereby inhibiting retinal NV (135). Oxidized LDL (oxLDL) has also been shown to elevate HIF-1α expression, and inhibition of HIF-1α blocks the angiogenic effect of oxLDL (136) (Figure 4D). Oxidative stress has also been implicated in the accumulation of HIF-1α in the RPE and the promotion of choroidal NV in age-related macular degeneration (AMD) (137). Collectively, these studies demonstrate how oxidative stress could contribute to the early induction of HIF-1α in DR, prior to the development of overt retinal ischemia.

### HIFs and inflammation in DR.

Accumulating evidence supports an interplay between HIFs and inflammatory mediators in the promotion of DR. IL-27 is significantly reduced in the aqueous humor of patients with DR compared with nondiabetic controls (138). IL-27 suppresses VEGF expression by reducing HIF-1α accumulation in macrophages from patients with DR (139). The expression of six-transmembrane epithelial antigen of the prostate 4, a membrane protein associated with hyperglycemic-induced inflammation, is decreased in human retinal vECs cultured in high glucose, preventing its ability to inhibit HIF-1α expression (140). A small peptide derived from the activity-dependent neuroprotective protein has been shown to prevent outer BRB breakdown by inhibiting HIF-1α and HIF-2α accumulation and, in turn, VEGF and VEGFR expression (141). The basal expression level of STING is stringently maintained by HIF-1α at the transcriptional level (142). STING also enhances NF-κB/HIF-1α/VEGF expression in oxidative stress–induced senescence of RPE (143) (Figure 4D). As HIFs have been reported to play a critical role in the inflammatory response in other conditions, further studies evaluating whether HIFs play a similar role in regulating the immune response in DR are warranted.

## Conclusion

Therapies targeting VEGF have had a remarkable impact on the treatment of patients with DR. Results from clinical trials assessing the efficacy of therapies targeting two other HIF-regulated genes, ANGPT2 and VE-PTP, have also shown promise. This has led to the development of a new class of therapies that target VEGF and ANGPT2 for the treatment of DME (144). An alternative approach is to target HIFs directly rather than the genes they regulate (Table 4). This approach takes advantage of the observation that HIFs increase expression of these factors primarily under pathological, not physiological, conditions (145). Another advantage of therapies targeting HIFs is that they reduce the expression of multiple vasoactive mediators to physiologic levels (107), and may play a similar role in regulating inflammatory mediators. This broad (arguably, more tempered) approach may mitigate the effects of completely neutralizing the expression of HIF-regulated vasoactive and inflammatory mediators while improving the efficacy of therapies targeting one (or two) vasoactive mediator(s).

Ongoing work from several labs has focused on repurposing available small molecule inhibitors to block HIF activity (105, 146). However, recent studies suggest that these drugs may not be suitable for treatment of ocular disease due to off-target effects causing toxicity to the neurosensory retina (107). This has led to the development of a new generation of HIF inhibitors. One example, 32-134D (147), was recently reported to be nontoxic to the retina at doses that effectively blocked HIF-1α and HIF-2α accumulation (107). Injection of 32-134D significantly inhibited retinal vascular hyperpermeability and retinal NV in mouse models of DM and AMD (107, 110, 148). Selective targeting of only HIF-2 with PT2385, a small-molecule HIF-2–specific inhibitor closely related to the recently FDA-approved drug belzutifan, also inhibited retinal NV in mouse models of ischemic retinopathies (103, 105). However, whether targeting HIF-2 alone will be sufficient for the treatment of patients with ischemic retinal disease remains unclear (105).

As with any target, it is important to acknowledge the potential limitations of inhibiting HIFs, which play an important protective role in cells and tissues in the setting of ischemic injury, oxidative stress, and inflammation. For example, pharmacologic HIF-1 inhibition increased, while HIF-1 augmentation decreased, photoreceptor apoptosis in two oxidative-stress mouse models, supporting a protective role for HIF-1 in photoreceptors in the setting of acute oxidative stress (137). Accordingly, stabilization of HIF-1α by pyruvate protected mouse photoreceptors against light-induced oxidative stress (149). HIFs also play a role in Müller cells and retinal neurons by coordinating cell metabolism, oxidative stress, and inflammation in DR. Consequently, nonselective HIF inhibition in DR could compromise critical neuroprotective and metabolic functions. Overcoming this limitation may require more refined therapeutic strategies, including cell-type-specific delivery platforms and temporal regulation of HIF activity, to preserve beneficial HIF functions while mitigating its pathogenic effects. Ultimately, understanding the relative contribution of HIFs and specific HIF-regulated genes to both the pathological and the protective response to retinal injury will be necessary to effectively design the next generation of therapies for DR.

## Funding support

This work is the result of NIH funding, in whole or in part, and is subject to the NIH Public Access Policy. Through acceptance of this federal funding, the NIH has been given a right to make the work publicly available in PubMed Central. The funding organizations had no role in the design or conduct of this research.

- NIH grants R01EY035889, R01EY029750, R01EY032104, and R21EY033934 to AS.
- Research to Prevent Blindness, Inc., Special Scholar Award (to AS) and Career Development Award (to CG).
- Norman Raab Foundation (to AS and CG).
- Branna and Irving Sisenwein Professorship in Ophthalmology (to AS).

## Figures and Tables

**Table 4:**
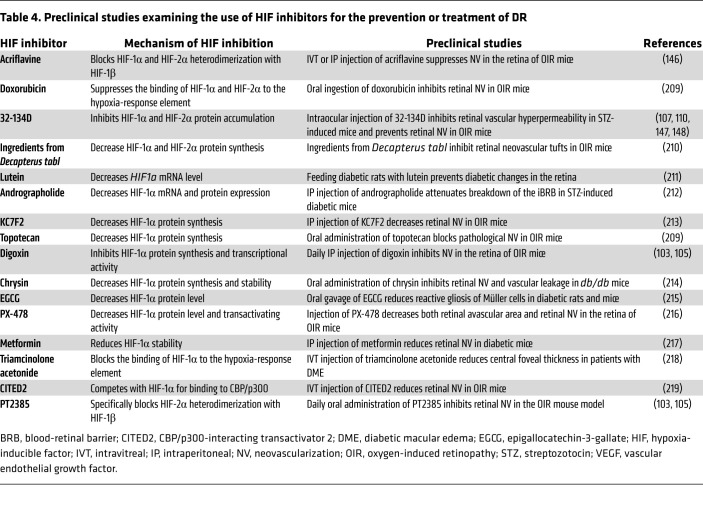
Preclinical studies examining the use of HIF inhibitors for the prevention or treatment of DR

**Table 1:**
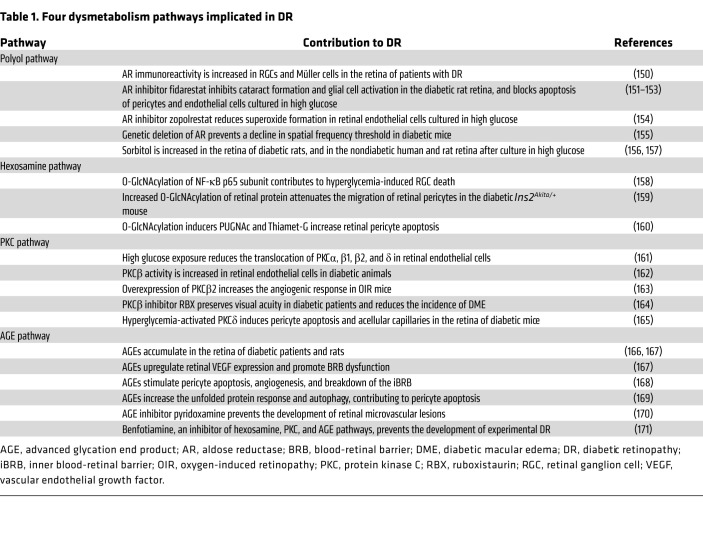
Four dysmetabolism pathways implicated in DR

**Table 2:**
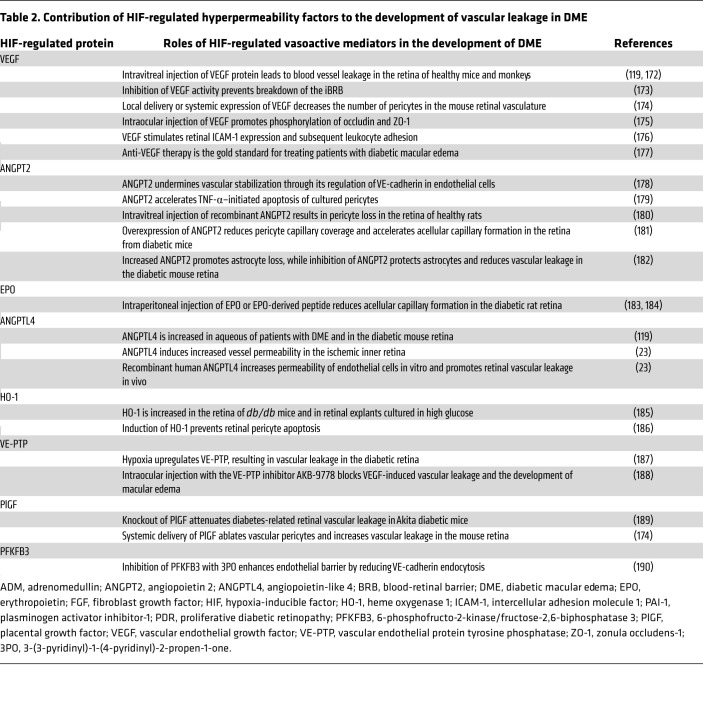
Contribution of HIF-regulated hyperpermeability factors to the development of vascular leakage in DME

**Table 3:**
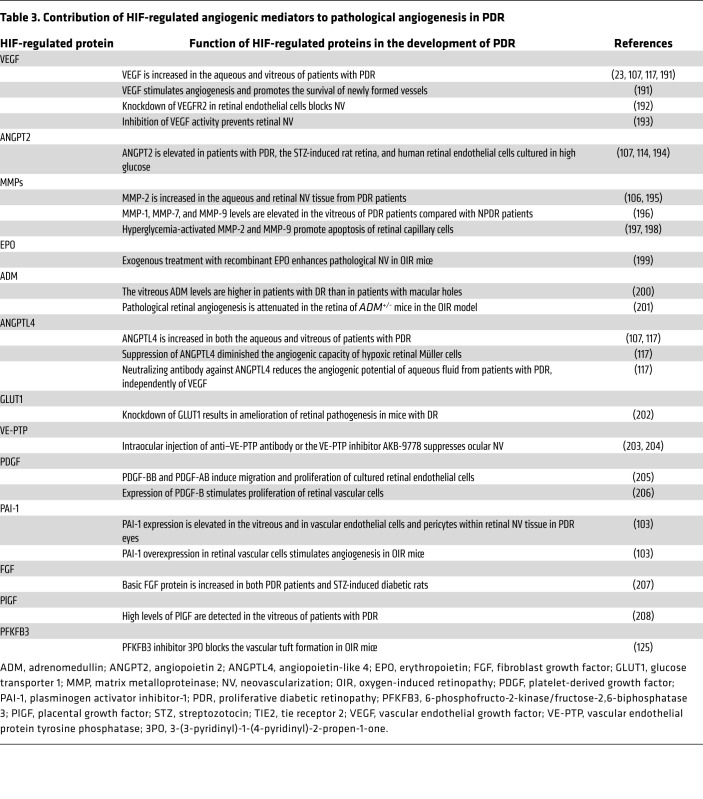
Contribution of HIF-regulated angiogenic mediators to pathological angiogenesis in PDR

## Version 1 03/02/2026

Electronic publication
